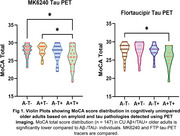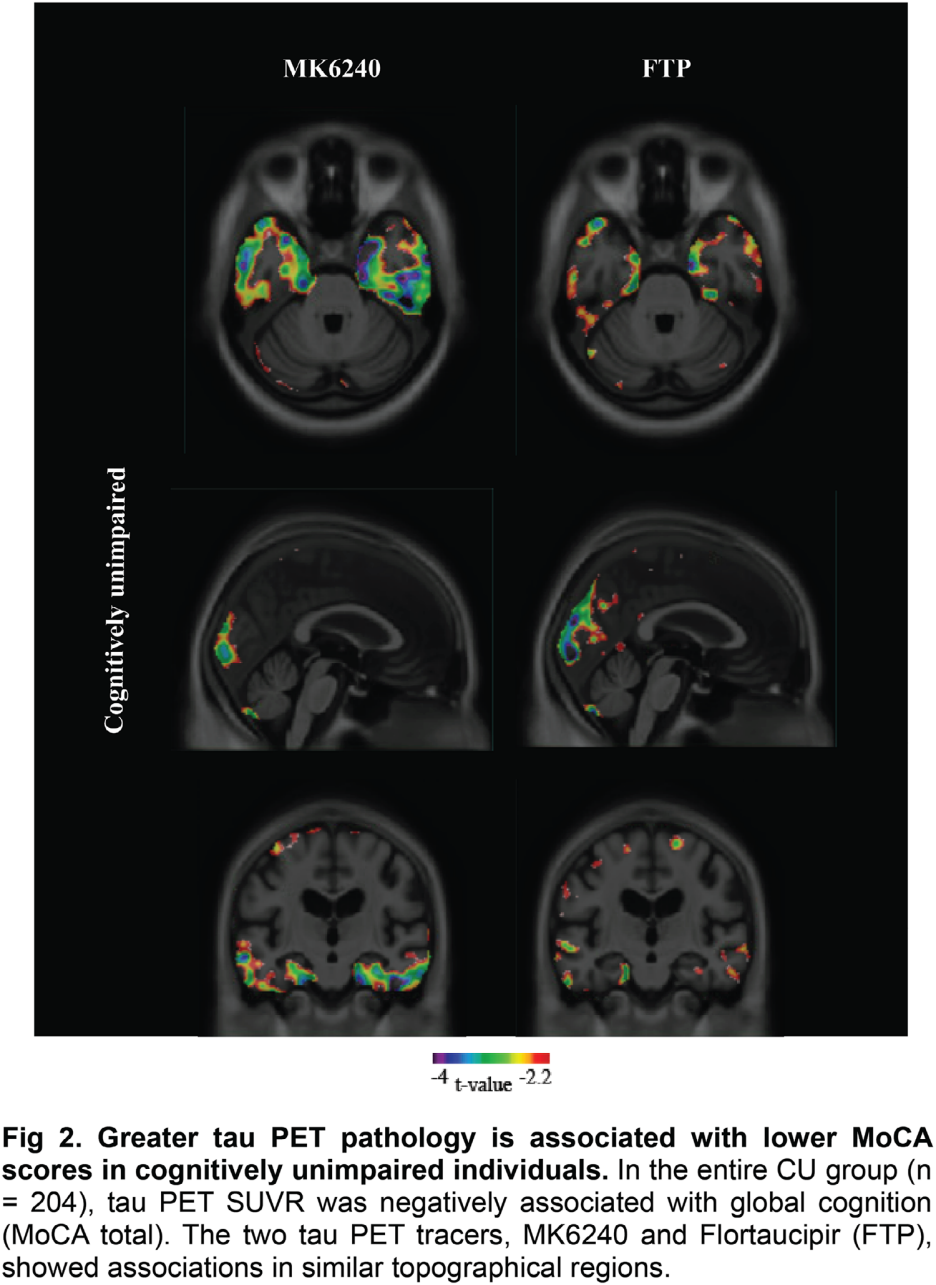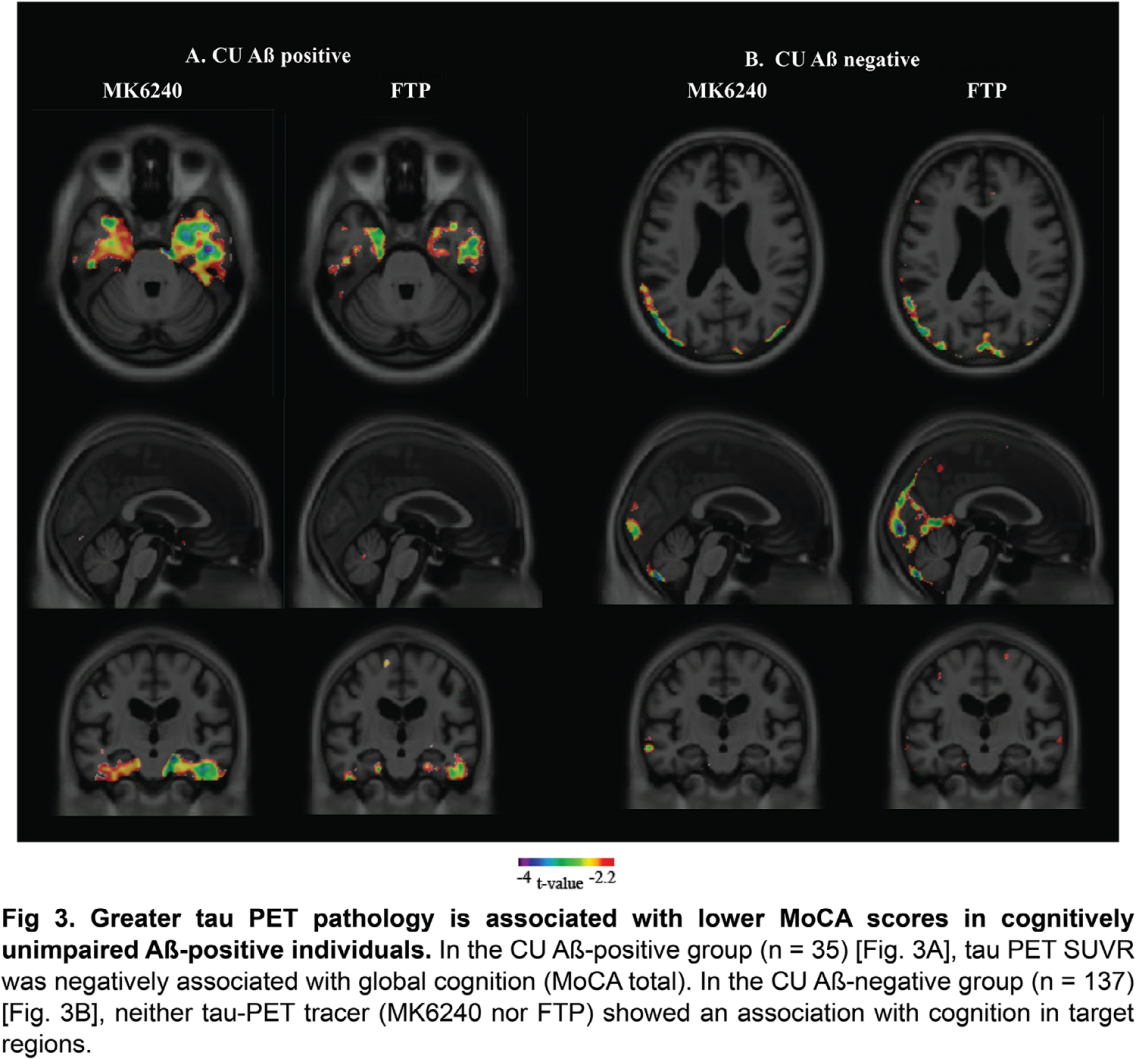# Usefulness of MoCA in detecting preclinical AD

**DOI:** 10.1002/alz70857_105912

**Published:** 2025-12-26

**Authors:** Cynthia Felix, Beth E. Snitz, Felix Joy Kollasserry, George Rebok, Pamela C.L. Ferreira, Dana L Tudorascu, Guilherme Povala, Pampa Saha, Livia Amaral, Firoza Z Lussier, Joseph C. Masdeu, Ziad Nasreddine, David N. Soleimani‐Meigooni, Juan Fortea, Val J Lowe, Hwamee Oh, Belen Pascual, Brian A. Gordon, Pedro Rosa‐Neto, Suzanne L. Baker, Tharick A Pascoal

**Affiliations:** ^1^ University of Pittsburgh, Pittsburgh, PA, USA; ^2^ University of Pittsburgh Alzheimer's Disease Research Center (ADRC), Pittsburgh, PA, USA; ^3^ Johns Hopkins University, Baltimore, MD, USA; ^4^ Houston Methodist Research Institute, Houston, TX, USA; ^5^ MoCA Clinic and Institute, Greenfield Park, QC, Canada; ^6^ Memory and Aging Center, Weill Institute for Neurosciences, University of California San Francisco, San Francisco, CA, USA; ^7^ Hospital de la Santa Creu i Sant Pau, Barcelona, Barcelona, Spain; ^8^ Mayo Clinic, Rochester, MN, USA; ^9^ Brown University, Providence, RI, USA; ^10^ Department of Radiology, Washington University in St. Louis, St. Louis, MO, USA; ^11^ Translational Neuroimaging Laboratory, The McGill University Research Centre for Studies in Aging, Montréal, QC, Canada; ^12^ Lawrence Berkeley National Laboratory, Berkeley, CA, USA

## Abstract

**Background:**

Individuals with preclinical AD are difficult to detect using traditional clinical tools. Yet, many individuals classified as cognitively unimpaired (CU) can exhibit a heterogeneous pattern of subtle clinical abnormalities, potentially linked to early Alzheimer's disease (AD). Primary care providers often have access only to clinical tests to study at‐risk community‐dwelling older adults who visit them without cognitive complaints. Since early intervention is crucial in AD, identifying clinical tools to detect preclinical AD is important in areas with limited access to blood or imaging biomarkers. In this study, we evaluate the link between Montreal Cognitive Assessment (MoCA) total scores and AD pathology in CU individuals.

**Methods:**

We studied 204 cognitively unimpaired (CU) older adults who underwent both ^18^F‐FTP and ^18^F‐MK6240 scans. These individuals were stratified based on their amyloid PET status, determined by visual reading, into CU A+ (*n* = 35) and CU A‐ (*n* = 137), as part of the ongoing HEAD study. CU adults had Clinical Dementia Rating (CDR) of 0 and were clinically identified as non‐MCI and non‐demented. Voxel‐wise linear regression models tested the association between MoCA total scores and tau pathology.

**Results:**

CU A+T+ had significantly lower total MoCA scores than A‐T‐ individuals (Figure 1). Lesser MoCA total score was significantly associated with greater mediobasal temporal tau deposition, using both ^18^F‐MK6240 and ^18^F‐FTP [Figure 2]. When we separated the population by Aβ status, we found that these results were driven by the CU A+ group (Figure 3A) and were not present in CU A‐ individuals (Figure 3B).

**Discussion:**

These findings indicate that the MoCA, a simple routine in‐office clinical test, can detect subtle cognitive dysfunction associated with preclinical AD. This suggests that simple cognitive testing can play a role in the early detection of AD and therefore an option for prescreening older adults in non‐specialized clinical settings, the initial interface for most older adults without cognitive complaints.

**Conclusion:**

MoCA total, the common dementia screening test also plays a valuable role in detecting preclinical AD.